# Tinea Imbricata among the Indigenous Communities: Current Global Epidemiology and Research Gaps Associated with Host Genetics and Skin Microbiota

**DOI:** 10.3390/jof8020202

**Published:** 2022-02-20

**Authors:** Yi Xian Er, Soo Ching Lee, Leslie Thian-Lung Than, Azdayanti Muslim, Kin Fon Leong, Zhenli Kwan, Izandis Mohd Sayed, Yvonne Ai-Lian Lim

**Affiliations:** 1 Department of Parasitology, Faculty of Medicine, Universiti Malaya, Kuala Lumpur 50603, Malaysia; eryixian@gmail.com; 2 Laboratory of Parasitic Diseases, National Institute of Allergy and Infectious Diseases, National Institutes of Health, Bethesda, MD 20892, USA; sooching.lee@nih.gov; 3 Department of Medical Microbiology, Faculty of Medicine and Health Sciences, Universiti Putra Malaysia, Serdang 43400, Malaysia; 4 Institute of Bioscience, Universiti Putra Malaysia, Serdang 43400, Malaysia; 5 Department of Medical Microbiology and Parasitology, Faculty of Medicine, Universiti Teknologi MARA (Sungai Buloh Campus), Sungai Buloh 47000, Malaysia; azdayantimuslim@gmail.com; 6 Institute for Medical Molecular Biotechnology (IMMB), Faculty of Medicine, Universiti Teknologi MARA (Sungai Buloh Campus), Sungai Buloh 47000, Malaysia; 7 Pediatric Institute, Kuala Lumpur General Hospital, Kuala Lumpur 50586, Malaysia; leongkinfon@gmail.com; 8 Division of Dermatology, Department of Medicine, Faculty of Medicine, Universiti Malaya, Kuala Lumpur 50603, Malaysia; zhenli@ummc.edu.my; 9 Hospital Orang Asli (Aborigines) Gombak, Jalan Pahang Lama, Gombak 53100, Malaysia; dr.izandis@moh.gov.my; 10 Centre for Malaysian Indigenous Studies, Universiti Malaya, Jalan 16/4, Seksyen 16, Petaling Jaya 46350, Malaysia

**Keywords:** tinea imbricata, *Trichophyton concentricum*, epidemiology, human genetics, skin microbiota

## Abstract

Tinea imbricata is a unique fungal skin disease that mostly affects indigenous populations in Southeast Asia, Oceania, and Central and South America. The control and management of this disease among these communities are challenging given their remote locations, certain traditional practices, and severe malnutrition status. To date, there are only a handful of reports published globally, which highlights the need for a more holistic approach in addressing this skin disease. Several bodies of evidence and reports have shown that host genetic factors have a profound influence on the pathogenesis of tinea imbricata, while skin microbiota is touted to have a role in the pathogenesis of the disease. However, there are limited studies of how host genetics and skin microbiota impact disease susceptibility in the host. To improve the understanding of this disease and to find possible long-term effective treatment among the affected indigenous communities, a comprehensive literature review is needed. Hence, this review paper aims to present the current status of tinea imbricata among the indigenous communities, together with published findings on the possible underlying reasons for its specific distribution among these communities, particularly on the ways in which host skin microbiota and host genetics affect occurrence and disease patterns. This information provides valuable insights for future research by highlighting the current knowledge gaps in these areas.

## 1. Introduction

**Figure 1:**
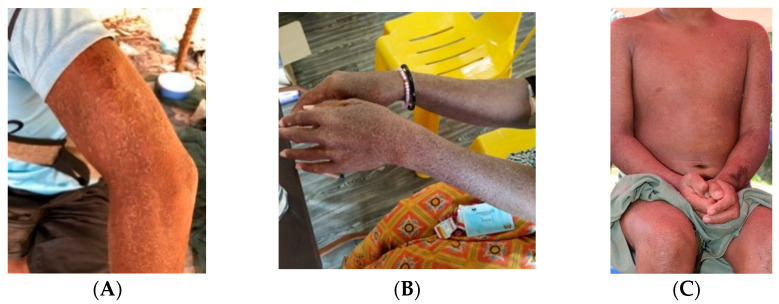
Various skin presentations of tinea imbricata among indigenous communities in Malaysia. (**A**) Patient from an indigenous background with tinea imbricata affecting the whole upper limb. (**B**) Signature concentric annular lesions on both upper limbs. (**C**) The annular lesions overlap each other to form a characteristic lamellar pattern. Photos (**A**,**B**) were taken with consent by Yi Xian Er in Kuala Koh, Kelantan, Malaysia and Baling, Kedah, Malaysia, respectively. Photo (**C**) was taken with consent by Dr. Kin Fon Leong.

Fungal skin diseases are among the most common diseases around the globe. They include tinea corporis (ringworm of the body), tinea capitis (ringworm of the scalp), tinea pedis (athlete’s foot), superficial candidiasis, and pityriasis versicolor. These skin diseases are caused by a variety of fungi, such as *Trichophyton, Epidermophyton*, *Microsporum*, *Malasezzia*, and *Piedrae hortae* [1]. Among the various types of tinea infections, one of the most intriguing is tinea imbricata, which is caused by *Trichophyton concentricum* [2]. The disease is well-known as it presents with unique circular lesions together with flaking of the skin, as shown in Figure 1A–C. Symptoms include pruritus, dry skin, and scaling of the skin. One of the unique characteristics of this disease is that it is mainly limited to indigenous communities in three geographical locations: Central and South America, Oceania, and Southeast Asia [2], with reports documented in Indonesia [3], the Solomon Islands [4], Malaysia [5], and Mexico [6]. The prevalence of this disease seems to be much higher in isolated and primitive societies [3]. The majority of patients in other regions reported travel history to these endemic zones and had close contacts with the local indigenous populations [7,8].

There are approximately 370 million indigenous people around the globe, across 90 countries, with more than 50% of them living in tropical areas [9]. They form at present non-dominant sectors of society and are determined to preserve, develop, and pass on to future generations their ancestral territories and their ethnic identity as the basis of their continued existence as peoples, in accordance with their own cultural practices, social institutions, and legal systems [10]. However, for the purpose of this article, we will only focus on the Austronesian people in Asia and Oceania and the Indigenous people of Central and South America, as they are the groups of people who are most affected by tinea imbricata.

Given their traditional practices and extreme and prolonged poverty [11], indigenous communities have higher risks of exposure to a variety of skin diseases. The control and management of tinea imbricata among indigenous communities is challenging because of their remote location, certain traditional practices [12], and severe malnutrition status [13]. The most recent case study showed that this disease still persists among indigenous communities [5], particularly among isolated subtribes, such as the Bateq subtribe in Peninsular Malaysia and the indigenous peoples in Papua New Guinea [14].

Several reports have been published globally highlighting the issue of the need for effective treatment agents and addressing the low treatment compliance among indigenous communities [15,16]. In general, the cure rate of skin diseases is largely determined by the patients’ adherence to treatment, which depends on factors such as treatment duration [17] and doses per day. Hence, fungal skin infections like tinea which typically require long treatment periods (up to a few months for tinea imbricata) can lead to low adherence by patients [18]. The challenges in the management of fungal skin infections are also exacerbated by the possible side-effects of medications, which include nausea and headache (i.e., terbinafine and griseofulvin) [19,20]. Moreover, remote locations and frequent recurrent infections exacerbate patients’ unwillingness to seek treatment.

Currently, there is no global prevalence data for tinea imbricata as the majority of the prevalence studies were regional. Most of the epidemiological studies date back to the 1990s or earlier. Most if not all of the latest publications were case reports rather than proper epidemiological studies. Given rapid global development in terms of infrastructure and economic advancement, the re-evaluation of the current global status of this disease especially among the indigenous population is vital.

In addition, the potential involvement of host genetics in the susceptibility of tinea imbricata has been reported in multiple publications. There seems to be an autosomal recessive gene associated with susceptibility to *Trichophyton concentricum*. Nevertheless, most of these studies were conducted in the 1980s or even earlier and there were limited genetic tools available during that time. Modern tools have been used to study other dermatophyte infections, such as those caused by *Trichophyton rubrum* [21] and *Trichophyton tonsurans* [22]. Hence, it is crucial to fill the knowledge gap of tinea imbricata susceptibility among indigenous populations with the aid of modern technology.

To achieve better understanding, treatment, and management of tinea imbricata, a more comprehensive investigation from the host perspective is necessary, particularly of the symbiotic relationship with host skin microbiota and with underlying host genetic factors. Skin microbiota is shaped by host genetics, diet, and living conditions [23,24,25]. Fungal skin diseases, such as tinea imbricata, cause drastic changes to the patients’ skin condition and topology. Host skin becomes dry with desquamation of large scales. It is hypothesised that this drastic change in host skin physiology appears to involve both the causative fungi and the skin microbiota. Although there have been no microbiota studies associated with tinea imbricata to date, there were studies among those with tinea pedis [26,27], which is caused by *Trichophyton rubrum*. Their research showed that there was variation in skin microbiota of patients and controls. However, the impact of these changes and the functions of these bacterial taxa are still under-studied and yet to be fully ascertained.

This present review highlights the current knowledge and epidemiology of tinea imbricata among indigenous peoples, together with the exploration of the possible underlying reasons of why it is largely confined among the indigenous communities. It is important to better understand the epidemiology and pathogenesis of this disease among these communities and to raise awareness among public agencies and relevant private entities for effective policies, strategies, and action plans to be instituted.

## 2. Materials and Methods

Multiple medical literature databases namely PubMed, Google, Google Scholar, and JSTOR were used to find relevant materials related to superficial mycoses, skin microbiome, host genetics, and indigenous people. Both layman and Medical Subject Headings (MeSH) terms were used in the search. The terms we searched included: indigenous/indigenous communities, Austronesian people, tinea imbricata, *Trichophyton concentricum*, human skin microbiome/microbiota, and host genotype/genes/genetic either singly or in combination. The search ceased once repetitive articles/unrelated articles were shown in the search results. Results in English, Malay, Indonesian, and Chinese languages were included in this review. We set a time frame of half a year to collect all the articles and materials. A total of 81 articles were included in this review.

Hence, the inclusion criteria were:

1. Written articles/materials in English, Malay, Indonesian, and Chinese languages, which include case reports, chapters of books, journal articles, news reports, meeting proceedings, unpublished theses and medical records.
2. No limitation on the publication date due to the scarcity of the data.
3. Articles need to have at least a correct description/photo of tinea imbricata (circular/lamella lesion, etc.).

Exclusion criteria were:

1. Materials written in other languages than English, Malay, Indonesian, and Chinese.
2. Materials such as video tape/photo footage.
3. Articles with incorrect description/photo of tinea imbricata.

## 3. Results

### 3.1. Introduction to Tinea Imbricata

#### 3.1.1. Taxonomy, Biology, and Microbiological Characteristics of *Trichophyton concentricum*

The causative agent of tinea imbricata, *Trichophyton concentricum*, is a strictly anthropophilic dermatophyte [28]. Based on the description by Reiss, 2011, the anamorph dermatophytes, including *Trichophyton concentricum*, are offshoots of the original teleomorphs, *Arthroderma benhamiae*. Over evolutionary time, the anamorph *Trichophyton concentricum* offshoots drifted away from their teleomorph ancestors. Phylogenetic analysis also indicated how *T. concentricum* is closely related to *A. benhamiae*, based on a genealogy analysis of actin, rRNA, and DNA topoisomerase 2 genes [29].

Information on the culturing of *T. concentricum* is scarce. Multiple reports suggested that it behaves similar to other dermatophytes and is capable of growing in Sabouraud glucose agar [2,7,30]. These reports showed that it is capable of growing in Mycosel agar (Sabouraud dextrose agar supplemented with cycloheximide and chloramphenicol) as well [8]. It produces smooth, waxy, and folded colonies on Sabouraud glucose agar [2,30]. The colonies are usually white-yellowish on top and yellow or tan under the surface, with a fine powdery down prominence on the edges [2]. The pigment might diffuse into the medium over time [2]. Aerial mycelium may develop in the aged fungal culture [2]. Some reports suggest that it can produce an amber colour and progressively darker colonies in 4% glucose agar [31].

Similar to other *Trichophyton* species, the microscopic appearance of *T. concentricum* consists of irregular branching hyphae and antler-like chlamydoconidia and macroconidia are rare, usally in lateral macroconidia, if present at all [2,30].

#### 3.1.2. Tinea Imbricata: Symptoms, Treatment Options, Outcomes, and the Challenges of Tinea Imbricata Treatment

Tinea imbricata usually starts with a rash in one site of the body, such as the abdomen [32] or knee [33]. The rash will then develop into concentric, ring-like lesions that will slowly progress and eventually cover a large body surface [2]. Some of the patients experience pruritus, especially during perspiration [34]. Relapse is common among susceptible groups [35]. Unfortunately, there have not been photomicrographs published in the available literature, although there was a publication which described the histopathological findings of tinea imbricata as acute and chronic inflammation with periodic acid–Schiff-positive fungal hyphae [36].

The treatment of the *T. concentricum* involves the usage of griseofulvin or terbinafine for a total of 3–4 weeks [5,7,19,37,38]; the dosage varies in accordance with the age of the patients, as shown in Appendix A Table A1. *T. concentricum* does not respond well toward the standard triazole anti-fungal drugs, such as itraconazole [19]. Other than itraconazole, *T. concentricum* also showed a much higher inhibitory concentration (22 mg/L) toward ketoconazole [2]. Treatment with oral antifungals may be combined with topical antifungal and keratolytics to improve the cure rate [37]. Among the indigenous people in endemic zones, such as Papua New Guinea, infected individuals wipe their lesions with *Cassia alata* leaves vigorously as part of the treatment [2], whereas indigenous people in the Philippines use the boiled extract of *Senna alata* leaves for treatment [38]. Remission usually will be seen after 3–4 weeks of the treatment. Nevertheless, it has been reported that 3–4% of the people might experience gastrointestinal effects when terbinafine is used [20].

Even with effective agents of treatment, challenges remain, as long treatment periods (3–4 weeks) often lead to low adherence and incomplete treatment [18]. In addition, most of the patients are indigenous people living in remote locations [5,16,38,39], which makes drug-delivery and patient follow-up difficult. Finally, due to the presence of the autosomal recessive gene [40], reinfection is likely as long as patients are living in the endemic zones [2], which might cause low adherence and confidence in the treatment offered. This leads to a vicious cycle which needs deeper understanding in order to offer better solutions to these communities.

### 3.2. Current Global Epidemiology of Tinea Imbricata among Indigenous People

Cases of tinea imbricata have been documented in Asia (i.e., Southeast Asia, South Asia, East Asia), Middle East, Oceania, and Central and South America, as illustrated in the following sections.

#### 3.2.1. Southeast Asia

**Malaysia**. Studies on tinea imbricata are scarce in Malaysia. Tinea imbricata is a well-known disease among locals as a disease that affects the indigenous populations. The term “kurap” probably referred to this disease in the early days [41]. The disease was first described in this country in an Englishman who acquired the disease after having close contact with an indigenous individual in 1952 [39]. This was followed by a prevalence study in the same year which reported the existence of the disease in four villages, with prevalence ranging between 1.7–18.3%. The study showed that the Lanoh people of the Negrito tribe had the highest prevalence (18.3%) compared to the Semai (9.9%) and Orang Seletar (4.4%) [42]. Two cases of tinea imbricata were reported in the *Lancet* journal involving two Caucasians who were a tin miner and a soldier, respectively, in the then Federation of Malaya. However, the sources of infections for these two cases were not mentioned besides a brief allusion that the two patients had been in Malaya for an extended period of time [43]. Subsequently, a report in 2002 mentioned that from 1993 to 2000, 20 (3.6% of the total isolates) cases of tinea imbricata at the University of Malaya Medical Centre (UMMC) were diagnosed among the indigenous people [44]. In 2018, a case report [5] documented this disease in an 8-year-old indigenous boy who lived in Gua Musang, Kelantan. More recently, there have been several reports highlighting this disease affecting a total of 120 villagers, constituting 80% of the population at Kampung Kuala Koh, Kelantan [15,16]. The most recent case report in 2022 was documented in a 4-year-old indigenous boy pinpointing that there were still cases of this disease in that area [45]. These studies indicate the persistence of this disease in the indigenous communities even after decades of the first report in 1952. Treatment challenges that have been highlighted include the remote locations of the patients’ villages and the low adherence to treatment [45].

**Indonesia**. Indonesia is another region where there have been reports of tinea imbricata, particularly in the indigenous communities around West Papua (formerly known as the Irian Jaya province). A prevalence study by Widianto in 1981 in Java showed low prevalence (i.e., 0.04%) [46]. However, studies in the less developed Kalimantan in 1995 showed that the prevalence ranged from 0.1–20.1%, which varies from one village to another, depending on the villages’ level of development [47]. A study in 1994 among 19 villages in Central Kalimantan showed that prevalence was around 2.5% or 150 cases among 6115 villagers [19]. Another report in 2013 [48] showed that the majority of the cases in Malawi, Kalimantan involved patients from the Dayak tribes (61%). Based on historical reports, tinea imbricata seemed to be very common in Sulawesi. “Even the King (“Raja”) there was reported to have suffered from skin diseases…”, and “Almost every native of Sangir and Talaud was afflicted by tinea imbricata…”, as quoted by the reports [49]. In addition, there was news of a 2-year-old boy who was diagnosed with tinea imbricata in Lombok, West Nusa Tenggara Province in 2018 [50]. There were case reports of tinea imbricata in a 47-year-old woman in 2016 [32] and a 28-year-old man in 2021 [14], both of whom were living in the Papua province.

**Philippines**. Tinea imbricata was common among the Tripoli tribes in the Philippines [36]. In fact, the earliest scientific description of tinea imbricata was documented in 1946 [51]. There was a case report in Mindoro Island [41] which involved a 52-year-old local who was treated with griseofulvin and calamine lotion. It was observed that locals in the Philippines used *Senna alata* decoction (concentrated leaf extract of the *Senna alata* plant) in the treatment of tinea imbricata. It was further reported that the decoction helped in reducing the average visual analogue scale (VAS) of pruritus by a score of 4.5 after 28 days treatment [34].

**Thailand**. The literature search showed only a single case report of tinea imbricata in Thailand in 1961, that of a 27-year-old male was treated successfully with fucin tablet (oral griseofulvin) for a total of 18 days. The patient then brought three of his family members from the village for treatment [52]. It is interesting to note that locals in Thailand call the disease “Hanumarn ringworm” as the patients’ skin resembled the appearance of Hanumarn, who is a monkey hero from a local ancient myth.

#### 3.2.2. South Asia

**India**. There have been reports of tinea imbricata in the Assam state of India. Some of the authors observed that this disease is quite common among the indigenous tribes in the hilly regions of the Assam state, particularly in the districts of Kamrup, Goalpara, and Nowgong [53]. Another case report featured an indigenous father and son from Darrang district, Assam state, both of whom were diagnosed with tinea imbricata in 1977 [54]. This disease is very rare among the non-indigenous communities in India. There was only one report of a male Bengali who contracted this disease due to frequent and close contact with the tribal people of Goalpara district, Assam during his childhood [55].

#### 3.2.3. East Asia

**China**. There have been records of tinea imbricata in China previously. However, since 2004, no case reports have been published. The last documented cases were detected in Shandong province [56]. The disease was called “Dié wǎ xuǎn” in China, which means “tinea which resembles the stacking roof tiles”. However, the case reports in China did not specify the ethnicities of the patients. Most of the reports were published between 1955–1970. It is interesting to note that cases in China were different to other endemic areas. Firstly, most of the cases were observed in male individuals (>90%) [57,58,59,60]. Secondly, these cases were reported as far as the north-east area of China (i.e., Liaoning province) [61], where the temperature and climate are quite the opposite of the tropics. Thirdly, cases in China demonstrated lesions which did not grow at thick keratinized areas, such as the feet and fingers [57]. Table 1 summarizes the case reports from China.

**Taiwan**. During the Japanese colonial era in Taiwan (1895–1945), cases were reported among the indigenous communities, such as Atayal and SaySiyat [62]. It was postulated that the disease was brought into Taiwan during the 14–17th centuries and it was described that the indigenous patients often suffered from circular and lamellar lesions that covered their entire bodies [52,62].

#### 3.2.4. Middle East

**Iran**. There was a case report in Iran involving a shepherd girl from Tavakkol Abad village, Kerman Province. This province has a warm and dry climate and low annual rainfall. In addition, the authors highlighted the poor hygiene and infrastructure of the village. Other associated factors highlighted in the report included malnutrition and unhygienic living conditions [63].

**Arabian Peninsula**. There was a report about a British soldier who was infected when he served in Arabia. The patient suffered from tinea imbricata since 1916. He was first admitted to the hospital in Aden, Yemen before being sent to a hospital in England for further treatment. The disease was still active till 1932 due to limited available drugs during that era. The authors mentioned the symptoms were reduced after fuchsin–acetone–resorcinol paint treatment was used [64].

#### 3.2.5. Oceania

Approximately 9–18% of the indigenous people in Oceania were affected by tinea imbricata [30]. Ambae and Bougainville islands of Vanuatu were known as the Île des Lepreux (Isle of Lepers) during the colonial era because the colonisers misinterpreted this locally endemic disease as leprosy [65].

**Papua New Guinea**. Tinea imbricata has existed in Papua New Guinea for a very long time. This was evident as some terms were developed due to this disease. Tribes would call patients with chronic tinea imbricata “pupuk”, which means ‘crocodile people’. These patients suffered from severe social disability due to marriage restrictions and segregation that was imposed on them [66]. Studies in 1963 [67] discovered that the prevalence of the disease in Maprik and Seprik districts ranged from 8–30% among the 5255 samples examined. Nutritional status was identified as an important factor. Areas with modern agricultural practices and growth had much lower prevalence. However, a report [68] in 1975 estimated the prevalence at 13.7% in Matang district among 802 samples examined. Similar to Schofield’s study, a variation in prevalence from 2.2–29.5% from one village to another village was observed as well. Another report showed a prevalence of up to 20% in Maprik district [69]. This disease was also found to be common in Kar Kar Island and Lufa district [70]. There was a rare case report in which a nurse who had constant close contact with local tribal people had erythematous lesions [33], unlike the locals. There was also evidence of griseofulvin usage to treat tinea imbricata, suggesting that this disease was widespread in that area [71,72].

**Fiji Island, the Solomon Islands, and New Zealand**. A survey in 1970 among 44 villages involving 5860 villagers revealed that the average prevalence of tinea imbricata was around 6% in Fiji Island. However, the prevalence was as high as 39% in certain villages [73] and was reported up to 20% in the Solomon Islands [30], while multiple case reports suggested that this disease has persisted among the indigenous populations in Fiji [74] and the Solomon Islands [75] up to the present day. Tinea imbricata has also been reported among New Zealand indigenous communities [76].

Most of the cases in Oceania were limited to indigenous communities; however, there were two documented cases among Italian tourists, who were infected in 2015 after having contact with the local tribal people in Tahiti and the Solomon Islands [7,8].

#### 3.2.6. Central and South America

Central and South American countries, such as Mexico and Brazil, are the other endemic zones of tinea imbricata. Similar to Southeast Asia, the majority of the cases are limited to local tribal people. Some researchers suggested that the disease was brought over by the Oriental immigrants during the pre-Columbian era [51]. There were many names given to the disease in this area, including “pita, tukune-tukune, oune, gorap, Kune-Kune, etc.” [40] These terms reflected the variety in native languages in that area and perhaps the history and the widespread distribution of tinea imbricata in that area as well.

**Mexico**. A report in 1976 [6] highlighted 16 cases of tinea imbricata (known as “cacapash” in the local language, meaning “full of scales”) in a mountainous indigenous village in Puebla, Mexico. The article also mentioned that the disease often presented during the rainy season and would resolve during the cold and dry season [6]. There was an article about an outbreak in a polygamous indigenous family of the Nahuatl zone in Mexico in which 9 out of 16 members had the disease [4].

**Brazil**. Tinea imbricata has been reported in Brazil as early as 1904 by Parahos and Leme [51]. However, we were unable to retrieve that manuscript as there was no scanned copy available online. There was a record [77] in 1982 of tinea imbricata diagnosed among native Indians in Xingu National Park of Brazil, but this disease was not common according to the authors. The latest case report in 2014 involved a 4-year-old indigenous child and his mother, suggesting that this disease is still present among the natives [35].

**Guatemala**. Tinea imbricata was reported in 1940 in Guatemala, where *Trichophyton concentricum* was successfully isolated from the patient [78]. Subsequently, it was reported among [51] 8 out of 211 members of a finca village in Solalá, Guatemala. The authors reported that the natives there live in close contact with their domestic animals, but the disease did not affect their domestic animals. The diet of the locals mainly consisted of beans and rice with small amounts of meat, and they rely on water with low iodine content (living in a goiter zone).

**Other countries in Central and South America**. There were cases reported in Columbia in 1904 by Mora Mora and El Salvador (1937 and 1938, respectively) [51]. Nevertheless, we were unable to review these papers further as there are no scanned/printed records in the database.

#### 3.2.7. Summary

**Figure 2:**
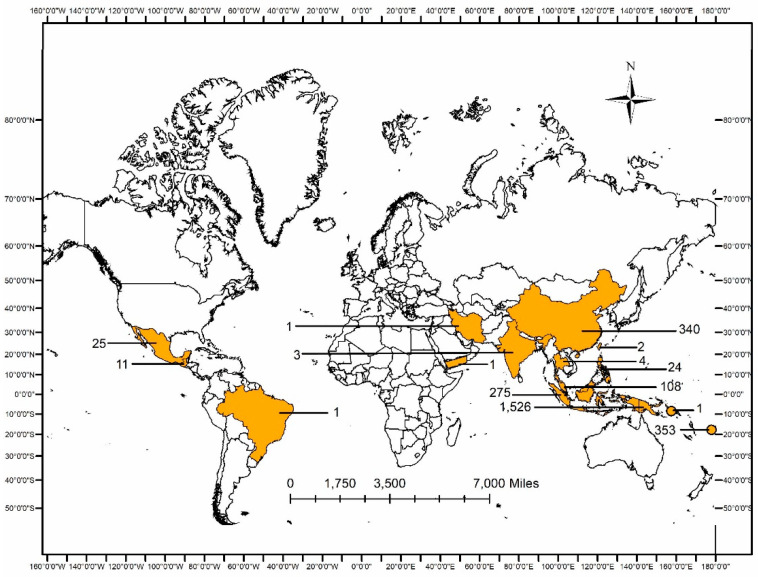
Number of reported cases of tinea imbricata around the globe, based on published articles.

Figure 2 shows the total number of available recorded cases around the globe based on collated published data. Most of the cases were found in Austronesian countries including Malaysia (*n* = 108), Indonesia (*n* = 275), Fiji (*n* = 353), and Papua New Guinea (*n =* 1526). Interestingly, there were quite a number of cases of tinea imbricata in China (*n* = 340) as well. Nevertheless, no ethnicity information was found in those records, hence it was uncertain whether the patients had any Austronesian origins or not. The cases in Central/South America (*n* = 37) seem low, but this is partly due to the fact that some of the published articles in the regions were in Spanish/Portuguese, which were not included in this review based on our inclusion criteria.

In brief, most tinea imbricata cases were reported among the indigenous communities. These cases tend to be more pronounced among villages which practice more primitive lifestyles and often have lower levels of hygienic practices. The non-indigenous people may acquire this disease from the indigenous people via close and constant contact. However, they have different prognoses compared to the indigenous people. The number of cases drops drastically when the level of urbanisation and education improve, such as in China and Java, Indonesia. A summary of patient characteristics, such as year, age, gender, clinical context, outcome, treatment, and sites of the well-recorded case reports, is provided in the supplementary section (Appendix B Table A2).

### 3.3. Tinea Imbricata and Host Genetics: Current Gaps

The persistence of tinea imbricata among indigenous communities has been associated with various factors, including under-developed living conditions and poor hygiene practices [2,3], thus leading to the persistence of *Trichophyton concentricum* spores in indoor air as well as on fomites, i.e., towels and bed sheets [79], posing a threat of infection. However, multiple articles have also described other related factors, such as geographical location, latitude, occupations [58,67], and host genetics [39]. This is not surprising as the disease is more prominent among isolated communities in which in-breeding might be common and, secondly, the majority of cases has been reported only among indigenous communities. Furthermore, one author mentioned that the patients suffered relapses after migrating out of the endemic area [35]. The weak dermal response of the patients toward *T. concentricum* suggests that the patients had different cellular-mediated immunity compared to others [4,35].

The involvement of host genetics was first suggested in 1963 [67]. In a survey of 4339 children between 0–15 years old, there were 36.8% children who did not develop symptoms despite living in the same household with both parents who were positive for tinea imbricata and having constant and close contact with the infected parents and siblings. In addition, the percentage of offspring with this disease dropped from 63.2% to 33.7% if only one of the parents was positive, and the percentage dropped further to 16.3% if neither parent was positive. However, the authors mentioned some sporadic cases in which local European or mixed-race descendants were infected, which means that no ethnic group was completely immune and that there were differing degrees of genetic susceptibility toward tinea imbricata [67].

Subsequently, a segregation analysis among 66 families in the Gogol Valley, Madang province of Papua New Guinea in 1977 [80] found that 16 out of 161 parents and 41 out of 399 offspring had tinea imbricata. These data were then applied to estimate the hypothetical recessive-gene frequency (0.3153). It was predicted that there will be 7 offspring with tinea imbricata from 54 families where both parents were positive, 4 offspring from 11 families where one of the parents was positive and only 1 offspring positive from 1 family when both parents were negative. The results showed that there was no statistically significant difference between the number of expected and observed offspring with tinea imbricata. The difference between expected outcome and observed outcome may be due to the inclusion of newborn children and the uncertainty of parentage in this population in which, as [67] noted, adultery was common.

The observation of Schofield et al. was further supported by a report in 1980 [39], in which a segregation analysis among 228 family pedigrees collected among villagers in Gogol Valley of Papua New Guinea was performed. The chi-squared test was used to test the randomness of mating and autosomal dominant inheritance followed by a priori segregation analysis to test the autosomal recessive inheritance. Their results supported the autosomal recessive inheritance of tinea imbricata and the recessive genes appeared to have unusually high frequency in the studied population (0.4889). Similar to a study in 1977 [80], this frequency was applied to predict the expected number of positive cases. The results showed no statistically significant difference between the expected and observed number of cases. These results suggested that the susceptibility to tinea imbricata is controlled by a recessive gene or genes segregating at a single autosomal locus. It was not biased towards any sex of the offspring, hence disproving sex-linked inheritance of the disease. The chi-squared test was also applied in this study to disprove the autosomal dominant inheritance of this disease.

In 1990, human leucocyte antigen (HLA) typing was carried out using restriction fragment length polymorphism (RFLP) among Melanesian indigenous people in Papua New Guinea [81]. Their results showed that the distribution of HLA-DRw16 was correlated with the distribution of tinea imbricata. However, there were no significant associations between HLA-DR genes and this disease. Interestingly, a study in 2004 suggested an autosomal dominant inheritance pattern of this disease among the natives in Mexico, though further verification would be needed as the study was conducted in only one family [4].

In summary, the association of tinea imbricata and host genetics seems apparent based on the variation in susceptibility among indigenous people and the limited distribution of this disease, namely, in the Caribbean [30], Oceania (up to 10–20% in the Solomon Islands [82]), and Southeast Asia (up to 9.8% in Malaysia [42]). In addition, studies in 1963, 1977, and 1980 indicated the plausibility that it is associated with autosomal recessive genes [42,69,82]. However, given the limited technology available during that time, the hypothesis could not be fully explored and confirmed, particularly in terms of the identification of the causative genes that are responsible for increased susceptibility. With current advancements in whole-genome sequencing and PCR technologies, detailed studies could be carried out to dive deeper into fungal skin infections and their associations with the human genome.

Based on the current trend, it is possible that more associations will be uncovered. With the advancement of modern technology, the discovery of the causative gene(s) of tinea imbricata should be achievable. These studies will lead to a different perspective in the treatment of skin diseases and may offer more permanent and effective solutions. Host genetics research has led to the understanding of risk factors underlying certain persistent infections, resulting in the modification and heightened effectiveness of current treatment protocols.

### 3.4. Tinea Imbricata and Skin Microbiota: Current Gaps

Another plausible reason for the variation in susceptibility to tinea imbricata is the variation in skin microbiota. Our skin is an intriguing and dynamic ecosystem that is inhabited by millions of microorganisms, including bacteria, archaea, fungi, and viruses. These microorganisms, collectively referred to as the skin microbiota, are crucial to skin physiology and immunity. Their interactions with humans fall anywhere along the continuum between mutualism and parasitism [83]. The advancement in PCR and high-throughput sequencing have unlocked the idea that skin microbiota may have a positive impact on the host.

There are multiple factors that affect the skin microbiota, which include diet [24], genetics [25], and the surrounding environment, such as the air and soil [23]. Hence, based on the same analogy, the isolated indigenous communities share a vastly different diet and living environment compared to non-indigenous people and tend to have their own unique sets of skin microbiota compared to the urbanites. This deduction was verified via recent research among Bolivian natives in the Amazon rainforest [84], where it was discovered that natives there had a much higher diversity of skin microbiota compared to urbanites.

Various articles have pinpointed the differences in the composition and function of the skin microbiota in patients with skin diseases because microbiota dysbiosis can lead to different host–microbe and also microbe–microbe interactions [85]. An article in 2013 showed that there was variation in the relative abundance of different bacteria in patients with atopic dermatitis and psoriasis with a significant increase in *Staphylococcus*, Propionibacteriaceae, and *Malassezia* [86]. These infections led to an increase in the overall abundance of certain microbes (i.e., *Staphylococcus* spp.) but, in turn, caused a decrease in overall microbiota diversity [85].

Skin microbiota dysbiosis has been observed in fungal skin infections as well, though all these studies focused on tinea pedis only and none on tinea imbricata (Table 2). Nevertheless, there are no definite answers as to whether dysbiosis makes the people vulnerable to dermatophytes or the skin barrier dysfunction due to infection then changes the microbiota.

In order to better understand how to manage and control tinea imbricata more effectively, research on the interaction between *T. concentricum* and the host is crucial. Unfortunately, there is a paucity of data on how *T. concentricum* impacts skin microbiota. Most of the studies among indigenous populations focused solely on gut and oral microbiomes [85,87,88]. These studies also showed that the indigenous populations harboured different types of microbiota (i.e., higher diversity) compared to the general population [84,89]. Therefore, research into this aspect will provide crucial knowledge which will lead to improved understanding of the pathogenesis of these diseases. However, there is no prior related research that has been carried out among indigenous communities. The relationship between tinea imbricata and skin microbiota is still unknown and needs to be elucidated.

## 4. Conclusions and Recommendations

This review provides useful insights into the most current epidemiological data on tinea imbricata among indigenous people. The prevalence remained under-reported for a long time even though it is among the most common dermatological conditions affecting isolated indigenous communities in tropical areas. Since the first report dating back to 1789 by Dampier [55], there has not been an updated and comprehensive study on the risk factors predisposing to this disease. Therefore, it is important to update the epidemiological data for tinea imbricata to establish effective management strategies in order to improve community health services provided by authorities, such as medical consultations and long-term plans, including monitoring for recurrent infections and the improvement of education and hygiene infrastructure.

Moreover, multiple reports suggested the involvement of host genetics in host susceptibility to tinea imbricata. Future studies evaluating the role of host genetics in this disease will be crucial in aiding prognostication. The discovery of host factors will allow the adoption of potential strategies (such as chitotriosidase therapy [87]) and reveal opportunities for the clinical translation of genetic information to the field of medical mycology. In the era of personalised medicine, the use of genetic data and individualised approaches may revolutionise the treatment and prevention of fungal diseases.

As the actual niche of skin microbiota in tinea imbricata is still under-researched, information on microbiota–pathogen interactions will provide useful knowledge in improving management of tinea imbricata and assist in improving the understanding of symbiosis between *T. concentricum* and host skin microbiota. In this context, the management of tinea imbricata and other related fungal skin infections may be more complicated than the management of other transmissible diseases. The persistence of the spores in the surroundings may propagate re-infection [79]. Hence, the microbiota may be the key component for a long-lasting solution for these diseases via bio-stimulation or microbial cocktail transplants.

## Figures and Tables

**Table 1:** Report of tinea imbricata in China.

Year	Reference	Description	Location
1954	[61]	12 cases in northern Jiangsu	Jiangsu Province
1958	[58]	106 cases from 1956–1957	Hefei City, Anhui Province
1960	[57]	26 cases recorded in Hospital of Shandong University between 1950–1956, which accounted for 0.1% of the total admitted cases at the Dermatology Department	Shandong Province
1960	[59]	183 cases recorded in a hospital in Shanghai	Shanghai
1963	[60]	12 cases in Anhui	Anhui province

**Table 2:** Summary of research on tinea pedis and skin microbiota.

Disease	Reference	Country	Sample Size	Description
Tinea pedis	[27]	China	29 patients, 14 patients (remission phase) and 36 controls	Patients showed lowest Shannon diversity of fungi communities and the highest diversity of bacteria among patients in remission compared to healthy controls.
	[26]	China	26 patients and 10 controls	Existence of variation in skin microbiota of patients with tinea pedis and controls. Skin microbial dysbiosis might impact the occurrence and the development of the disease.

## Appendix A

**Table A1:** Dosage of griseofulvin and terbinafine in the treatment of tinea imbricata.

Reference	Drugs	Category	Dosage	Note
[88]	Griseofulvin	Adult	0.500–1.000 g/day	Dosage varies depending on the total area of the lesions
		Paediatrics	0.125–0.250 g/day	14–23 kg
			0.250–0.500 g/day	>23 kg
[90]	Terbinafine	Adult	0.250 g/day	-
		Paediatrics	0.0625 g/day	10–20 kg
			0.125 g/day	20–40 kg
			0.250 g/day	>40 kg

## Appendix B

**Table A2:** Compilation of the collected case report of tinea imbricata around the globe.

Region	Country	Citation	Year	Gender	Ethnicity	Age (Years)	Clinical Manifestation	Treatment	Outcome
South East Asia	Indonesia	[32]	2016	Female	Melanesian	47	Concentric rings developed in the abdomen and eventually the lesions grew and covered the whole body except the face, patient experienced desquamation and severe pruritus.	2% ketoconazole cream (2 times/day) for a total of 9 weeks, oral 200 mg ketoconazole 1 time/day for a total of 6 weeks, oral 4 mg chlorphenamine/day for the first 2 weeks of treatment.	Reduction of the concentric lesions and hyperpigmentation occurred starting from day 28 and the full remission occurred on day 56.
		[14]	2021	Male	Melanesian	28	Concentric erythematous lesions with thick scales over most of the body surface.	500 mg of oral griseofulvin 2 times/day for a total of 15 weeks, oral cetirizine 10 mg/day, topical miconazole 2% cream 2 times/day, topical 3% salicylic acid mixed with 10% urea cream twice/day.	Potassium hydroxide examination and cultures were negative after 15 weeks of treatment.
	Malaysia	[41]	1952	Male	Caucasian	23	Pleomorphic eruption on the limbs and chest, easily-detached skin scales.	Two topical drugs were used twice daily to the whole body for alternate weeks, treatment duration of a few weeks: (1) Whitfield’s ointment (2) Lotion which contained: salicylic acid, 2 drachms (30 gm); copper nitrate (0–5 gm); acetone (25 mL); methylated spirit (100 mL).	Remission observed after 9 weeks, only minor lesions observed.
		[43]	1962	Male	Caucasian	42	Annular eruption in the upper limbs initially, followed by redness, scaling and annular lesions that covered the whole body in the later stage.	500 mg of griseofulvin, 3 times/day for 3 weeks, then reduced to two times/day for another 3 weeks.	Remission after 6 days of treatment.
				Male	Caucasian	45	Started with rash on the back of his arm, followed by concentric rings of scales that covered the whole body.	500 mg of griseofulvin, 2 times/day for a total of 3 weeks. He received an additional 2 weeks of treatment after 4 months due to a relapse.	Remission observed after 8 days. The relapse was observed in the right thigh, 4 months after the first therapy. No further relapse observed after the second treatment.
		[5]	2018	Male	Indigenous	8	Round, concentric, and scaly rash that covered 80% of the body surface, experienced itch.	125 mg of oral terbinafine for 8 weeks.	Complete clearance of the lesion after treatment.
		[45]	2022	Male	Indigenous (Bateq)	4	Concentric, annular skin lesions which started from the abdomen and spread to upper limbs and face, experienced severe pruritus during hot weather.	Syrup griseofulvin 10 mg/kg daily and Whitfield cream for 4 weeks.	Patient discharged 6 days after treatment, skin condition improved at follow-up after a month.
	Philippines	[34]	1962	Male	Not disclosed	52	Erythematous maculopapular eruption on the trunk and extremities, skin was thin and flaky, and lesions were arranged in polymorphic and polycyclic concentric rings. The patient complained of pruritus particularly when perspiring.	250 mg of oral griseofulvin for a total of 4 times/day together with calamine lotion to ease the itch for a total of 5 weeks.	Improved on the 6th day of the treatment and complete remission was observed on the 14th day.
		[36]	2010	Male	Indigenous	30	Polycyclic to serpiginous scaling lesions.	500 mg of oral griseofulvin daily.	No follow-up recorded.
			2010	Female	Indigenous	40			
			2010	Female	Indigenous	19			
	Thailand	[52]	1961	Male	Not disclosed	27	Generalised pruritus initially, followed by the development of concentric rings that covered the body within 3 months.	Oral griseofulvin 500 mg 4 times/day for the first 2 days, then the dosage reduced to 2 times/day for the next 7 days, and then further reduced to 250 mg 3 times/day and nocte for 9 days.	Pruritus reduced on the 7th day of the treatment and microscopic results were negative on the 10th day. No relapse occurred 6 months after treatment.
South Asia	India	[54]	1977	Male	Nepali	20	Scaly, itchy lesions all over the body since he was young. The lesion started from his forearm and gradually involved the entire body.	250 mg of oral griseofulvin for a total of 2 times/day together with high fatty meals for 60 days.	Marked improvement on the 4th day of the treatment, clinical clearance after a month of treatment and microscopic results were negative on the 60th day of the treatment.
East Asia	China	[60]	1963	Male	Not disclosed	31	Concentric lesions on face and limbs.	500 mg of oral griseofulvin per day for a total of 3 weeks.	Relapse occurred 1 month after treatment.
				Male		24	Hand-size lesions on face, thigh, right arm, and knee.	500 mg of oral griseofulvin per day for a total of 23 days	Relapse occurred 2 weeks after treatment.
				Male		41	Concentric lesions that covered most of the body surface.	500 mg of oral griseofulvin per day for a total of 3 weeks.	Relapse occurred 10 days after treatment.
				Male		24	Concentric lesions that covered the scalp, back, thigh, and right forearm.	500 mg of oral griseofulvin per day for a total of 3 weeks.	No follow-up was done.
				Male		34	Concentric lesions that covered most of the body surface.	500 mg of oral griseofulvin per day for a total of 26 days.	Relapse occurred 5 months after treatment.
				Male		26	Concentric lesions that covered most of the body surface.	1000 mg of oral griseofulvin per day for a total of 4 weeks.	No follow-up was done.
				Male		24	Concentric lesions that covered the face, neck, chest, back, both arms, and right thigh.	1000 mg of oral griseofulvin per day for a total of 2 weeks	Relapse occurred 2 months after treatment.
				Male		39	Concentric lesions that covered most of the body surface.	1000 mg of oral griseofulvin per day for a total of 32 days.	Relapse occurred 1 year after treatment.
				Male		32	Concentric lesions on the right ear, both cheeks, and thighs.	1000 mg of oral griseofulvin per day for a total of 4 weeks.	Relapse occurred 2 months after treatment.
				Male		37	Concentric lesions on both ears, right upper limb, both lower limbs, and groin area.	1000 mg of oral griseofulvin per day for a total of 36 days.	Relapse occurred 4 months after treatment.
				Male		47	Concentric lesions on both ears, 4 limbs, and the abdomen.	1000 mg of oral griseofulvin per day for a total of 4 weeks, then 1500 mg of oral griseofulvin per day for a total of 41 days to treat a relapse, which was 62 days after the first treatment.	Relapse occurred 3 months after the second treatment.
				Male		35	Concentric lesions on the scalp, both ears, neck, and face.	1500 mg of oral griseofulvin per day for a total of 37 days.	No relapse occurred within a 3-month follow-up period after treatment
				Male		41	Concentric lesions that covered most of the body surface.	1500 mg of oral griseofulvin per day for a total of 29 days.	Relapse occurred 3–4 months after the second treatment.
Middle East	Iran	[63]	2009	Female	Not disclosed	10	Hypopigmented circular lesions on wrist and forearm, pruritus.	250 mg of oral terbinafine per day, topical clotrimazole 1% ointment and miconazole 2% cream twice daily, potassium permanganate for daily washing. Treatment lasted for a total of 4 weeks.	Treated successfully and no report of relapse.
	Yemen	[64]	1932	Male	Caucasian	Not disclosed	Eruption covered most of the body parts, scaling of skin observed.	Fuchsin–acetone–resorcinol paint was used, duration not disclosed.	Condition improved after treatment.
Oceania	Papua New Guinea	[33]	1988	Female	Caucasian	23	A lesion with 10 cm diameter in the antero-lateral aspect of the left knee, showed a peripheral, serpiginous ring of erythema, and scaling.	1000 mg of oral griseofulvin per day for 4 weeks.	Experienced diarrhoea and abdominal discomfort due to griseofulvin in the first week of the treatment. Skin was clinically and mycologically clear after ten days. No relapse after 2 months, though hypopigmentation remained around the lesion.
	Fiji	[74]	2016	Female	Melanesian	18	Concentric, scaly rash that covered 70% of the body surface.	Oral griseofulvin and dilute vinegar soaks to prevent relapse.	No follow-up due to the remote location of the patient
	Solomon Island	[8]	2015	Male	Caucasian	6	Multiple annular, concentric, squamous lesions on right upper limb and back; mild pruritus.	Griseofulvin (10 mg/kg/day) and 1% terbinafine cream (2 times/day) for 6 weeks.	Complete remission after the treatment and no relapse during follow-up (2 months after the treatment).
	Tahiti	[7]	2015	Female	Caucasian	47	Multiple, annular, concentric, erythematous-purpuric lesions on the buttocks, thigh, right breast, abdomen, and legs.	Griseofulvin (10 mg/kg/day) and 1% terbinafine cream (2 times/day) for 6 weeks.	Clinically negative but mycologically positive at third week of the treatment. Complete remission after treatment, no relapse during follow-up (12 months after treatment).
South America	Brazil	[35]	2014	Male	Brazilian	2	Itchy and centrifugally growing lesion on the face.	5 weeks of griseofulvin treatment.	Clinically and mycologically negative after treatment, no relapse during the next 6 months.

(Only case reports that provided information on country, regions, year as well as sufficient patient information (gender, ethnicity, symptoms, treatment, and outcome) were included).

## References

[1] Schwartz R.A. (2004). Superficial fungal infections. Lancet.

[2] Hay R.J. (1988). Tinea imbricata. Curr. Top. Mycol..

[3] Bramono K. (2012). Chronic Recurrent Dermatophytosis in the Tropics: Studies on Tinea imbricata in Indonesia. Korean J. Med. Mycol..

[4] Bonifaz A., Araiza J., Koffman-Alfaro S., Paredes-Solis V., Cuevas-Covarrubias S., Rivera M.R. (2004). Tinea imbricata: Autosomal dominant pattern of susceptibility in a polygamous indigenous family of the Nahuatl zone in Mexico. Mycoses.

[5] Leung A.K., Leong K., Lam J.M. (2018). Tinea imbricata. J. Pediatr..

[6] Velasco-Castrejón O., González-Ochoa A. (1976). Tinea imbricata in the mountains of Puebla, Mexico. Rev. Investig. Salud Pública.

[7] Veraldi S., Pontini P., Nazzaro G. (2015). A case of tinea imbricata in an italian women. Acta Derm. Venereol..

[8] Veraldi S., Giorgi R., Pontini P., Tadini G., Nazzaro G. (2015). Tinea Imbricata in an Italian Child and Review of the Literature. Mycopathologia.

[9] Buchholz K. Where the World′s Indigenous People Live. https://www.statista.com/chart/18981/countries-with-the-largest-share-of-indigenous-people/.

[10] Coates K.S. (2004). A Global History of Indigneous People.

[11] Lin K.G., Zalilah M.S. (2008). The Ecology of Health and Nutrition of “Orang Asli”(Indigenous People) Women and Children in Peninsular Malaysia. Tribes Tribals.

[12] Collins N. Health Care for the Orang Asli: Consequences of Paternalism and Colonialism. https://www.coac.org.my/main.php?section=articles&article_id=13.

[13] Singh A. (2008). Mortality, Morbidity and Malnutrition among Orang Asli Children.

[14] Wijaya H., Ratih N.L.P., Karna V. A Rare Case of Tinea Imbricata. Proceedings of the Congress of ISHAM Asia Fungal Working Group.

[15] Zahari B.J. (2019). Batek Tribe Told to Seek Immediate Treatment for Fungal. New Straits Times.

[16] Ibrahim R. (2019). Penduduk Kampung Aring 5 Pula Dijangkiti Penyakit Kulit. Metro.

[17] Lee J.M., Shun D.H., Choi J.S., Kim K.H. (1998). Analysis of Treatement Results of patients with Tinea Unguium and Assessment of the Real Effectiveness of Antifungal Agents and Patient Compliance. Korean J. Dermatol..

[18] Weinberg J.M. (2009). Increasing Patient Adherence in Antifungal Infection Treatment: Once-Daily Dosing of Sertaconazole. J. Clin. Aesthetic Dermatol..

[19] Budimulja U., Kuswadji K., Bramono S., Basuki J., Judanarso L.S., Untung S., Widago S., Rhprabowo W., Koesanto D., Widjanarko P. (1994). A double-blind, randomized, stratified controlled study of the treatment of tinea imbricata with oral terbinafine or itraconazole. Br. J. Dermatol..

[20] Villars V., Jones T.C. (1989). Clinical efficacy and tolerability of terbinafine (Lamisil)—a new topical and systemic fungicidal drug for treatment of dermatomycoses. Clin. Exp. Dermatol..

[21] Jaradat S.W., Cubillos S., Krieg N., Lehmann K., Issa B., Piehler S., Wehner-Diab S., Hipler U.-C., Norgauer J. (2015). Low DEFB4 Copy Number and High Systemic hBD-2 and IL-22 Levels Are Associated with Dermatophytosis. J. Investig. Dermatol..

[22] Abdel-Rahman S.M., Preuett B.L. (2012). Genetic Predictors of Susceptibility to Cutaneous Fungal Infections: A pilot Genome Wide Association Study to Refine a Candidate Gene Search. J. Dermatol. Sci..

[23] Selway C., Mills J.G., Weinstein P., Skelly C., Yadav S., Lowe A., Breede M.F., Weyrich L.S. (2020). Transfer of environmental microbes to the skin and respiratory tract of humans after urban green space exposure. Environ. Int..

[24] Brandwein M., Katz I., Katz A., Kohen R. (2019). Beyond the gut: Skin microbiome compositional changes are associated with BMI. Hum. Microbiome J..

[25] Si J., Lee S., Park J.M., Sung J., Ko G. (2015). Genetic associations and shared environmental effects on the skin microbiome of Korean twins. BMC Genom..

[26] Liu X., Tan J., Yang H., Gao Z., Cai Q., Meng L., Yang L. (2019). Characterization of Skin Microbiome in Tinea Pedis. Indian J. Microbiol..

[27] Wang R., Song Y., Du M., Yang E., Yu J., Wan Z., Li R. (2018). Skin microbiome changes in patients with interdigital tinea pedis. Br. J. Dermatol..

[28] Reiss E., Shadomy H.J., Lyon G.M. (2011). Fundamental Medical Mycology.

[29] Kawasaki M., Anzawa K., Wakasa A., Takeda K., Tanabe H., Mochizuki T., Ishizaki H., Hemashettar B.M. (2008). Different Genes Can Result in Different Phylogenetic Relationships in Trichophyton Species. Nippon Ishinkin Gakkai Zasshi.

[30] Bonifaz A., Archer-Dubon C., Saúl A. (2004). Tinea imbricata or Tokelau. Int. J. Dermatol..

[31] Castellani A. (1913). Note on the Ætiology of Some Tropical Dermatomycoses. Proc. R. Soc. Med..

[32] Johan R. (2016). Tinea Imbrikata. Cermin Dunia Kedoktoran.

[33] Logan R.A., Kobza-Black A. (1988). Tinea imbricata in a British nurse. Clin. Exp. Dermatol..

[34] Fernandez M.C. (1962). Tinea Imbricata Successfully Treated with Griseofulvin. Arch. Dermatol..

[35] Jiménez L.M., Monsálvez V., García-Rodrigo C.G., Llorente C.P. (2014). Tinea Imbricata as a Clue to Occult Immunodeficiency. Pediatr. Dermatol..

[36] Non L.B.R., Dofitas B.L. (2010). Tinea Imbricata: Case series on three patients in Sarangani, Philippines. Acta Med. Philipp..

[37] Leung K.C.A., Leong F.K., Lam M.J. (2019). Tinea Imbricata: An Overview. Curr. Pediatr. Rev..

[38] Eusebio-Alpapara K.M.V., Dofitas B.L., Balita-Crisostomo C.L.A., Tioleco-Ver G.M.S., Jandoc L.E., Frez M.L.F. (2020). *Senna (Cassia) alata* (Linn.) Roxb. leaf decoction as a treatment for tinea imbricata in an indigenous tribe in Southern Philippines. Mycoses.

[39] Sharvil D. (1952). Tinea imbricata in a European: Double infection with Trichophyton concentricum and Trichophyton rubrum. Br. J. Dermatol..

[40] Ravine D., Turner K.J., Alpers M.P. (1980). Genetic inheritance of susceptibility to tinea imbricata. J. Med. Genet..

[41] Benjamin G. (1968). Temiar Personal Names. J. Humanit. Soc. Sci. Southeast Asia Ocean..

[42] Polunin I. (1952). Tinea imbricata in Malaya. Br. J. Dermatol..

[43] Church R., Sneddon I. (1962). Tinea imbricata—A report of two cases treated with Griseofulvin. Lancet.

[44] Ng K.P., Soo-Hoo T., Na S., Ang L. (2002). Dermatophytes isolated from patients in University Hospital, Kuala Lumpur, Malaysia. Mycopathologia.

[45] Roslan S.R., Hadi A.A. (2022). A child with unique skin pattern: A case report of Tinea imbricata. Med. J. Malays..

[46] Widyanto (1981). Cases Study of Tinea Imbricata in Mauk.

[47] Budimulja U. (1995). Tinea imbricata in central Kalimantan. Malays. J. Dermatol..

[48] Gerry G. (2013). Gambaran Distribusi Penderita Penyakit Tinea Imbrikata Berdasarkan Keadaan Sosiodemografi dan Klinis di Desa Teluk Pongkal Kecamatan Sokan Kabupaten Melawi Provinsi Kalimantan Barat Tahun 2010. J. Mhs. Fak. Kedokt. Untan.

[49] Henley D., Van Dijk C., Taylor J.G. (2011). Hygiene, housing and health in colonial Sulawesi. Cleanliness and Culture.

[50] ACTNews R. Abdan, Bocah Penderita Tinea Imbrikata dari Lombok Utara. https://news.act.id/berita/abdan-bocah-penderita-tinea-imbrikata-dari-lombok-utara.

[51] Gomez J.E. (1946). Tokelau in Guatemala. Arch. Dermatol..

[52] Chermsirivathana S., Boonsri P. (1961). A case of tinea imbricata (Hanumarn ringworm) treated with fulcin. Aust. J. Dermatol..

[53] Dey N.C., Maplestone P.A. (1942). Tinea Imbricata in India. Indian Med. Gaz..

[54] Das J., Kukreja B. (1977). Tinea Imbricata. Indian J. Dermatol. Venereol. Leprol..

[55] Acton H.W., Ghosh L.M. (1934). Tinea Imbricata (Tokelau) in Bengal. Indian Med. Gaz..

[56] Li Z.-X., Li S.-D. (2004). Tinea imbricata (1 case). China J. Lepr. Ski. Dis..

[57] Liu C.-L. (1960). Analysis of 26 cases of tinea imbricata. Chin. J. Dermatol..

[58] Zhu Y.-Y. (1958). Analysis of 106 cases of tinea imbircata in Hefei City. Chin. J. Dermatol..

[59] Qing Q.-X., Huang Z.-J., Yuan C.-Y. (1960). Clinical observation and laboratory inspection of 183 cases of tinea imbricata. Chin. J. Dermatol..

[60] Mao C.-B., Zhou Y.-D., Wong G.-S., Zheng M.-R. (1963). Treatment of tinea imbricata and tinea with griseofulvin (Report of a total of 15 cases). Chin. J. Dermatol..

[61] Zhou Y.-D. (1954). Observation of 12 cases of tinea imbricata. Chin. J. Dermatol..

[62] Chang H.-J. (2015). 50 Years of Advancement: A Collection of Taiwan’s Medical and Public Health Records under the Japanese Colonial Rule.

[63] Mousavi S.A.A., Sardoii S.S., Shamsadini S. (2009). A first case of tinea imbricata from Iran. Jundishapur J. Microbiol..

[64] Castellani A. (1932). Tinea Imbricata in a European. J. R. Soc. Med..

[65] Jolly M., Tcherkézoff S., Tryon D. (2009). The Sediment of Voyages: Remembering Quirós, Bougainville and Cook in Vanuatu. Oceanic Encounters.

[66] Cockburn T.A. (1971). Infectious diseases in ancient populations. Curr. Anthropol..

[67] Schofield F.D., Parkinson A.D., Jeffrey D. (1963). Observations on the epidemiology, effects and treatment of Tinea imbricata. Trans. R. Soc. Trop. Med. Hyg..

[68] MacLennan R., O′Keeffe M. (1975). Altitude and prevalence of tinea imbricata in New Guinea. Trans. R. Soc. Trop. Med. Hyg..

[69] Plato C.C., MacLennan R. (1975). The dermatoglyphics of the Maprik sub-district of the Sepic district of New Guinea. Z. Morphol. Anthropol..

[70] Hornabrook R.W., Serjeantson S., Stanhope J.M. (1977). The Relationship between Socioeconomic Status and Health in Two Papua New Guinean Populations. Hum. Ecol..

[71] Dresser C.K. (1964). Fine Particle Griseufulvin in tinea imbricata. Dermatologica.

[72] MacLennan R. (1960). A trial of griseofulvin in tinea imbricata. Trans. St. John’s Hosp. Dermatol. Soc..

[73] Ajello L. (1972). The mycoses of Oceania. Mycopathol. Mycol. Appl..

[74] Burns C., Valentine J. (2016). Tinea Imbricata. N. Engl. J. Med..

[75] Pihet M., Bourgeois H., Mazière J.-Y., Berlioz-Arthaud A., Bouchara J.-P., Chabasse D. (2008). Isolation of Trichophyton concentricum from chronic cutaneous lesions in patients from the Solomon Islands. Trans. R. Soc. Trop. Med. Hyg..

[76] Rush-Munro F.M. (1980). Tinea imbricata in New Zealanders. N. Z. Med. J..

[77] Baruzzi R.G., Marcopito L.F., Vicente L.S., Michalany N.S. (1982). Jorge Lobo′s Disease (Keloidal Blastomycosis) and Tinea Imbricata in Indians from the Xingu National Park, Central Brazil. Trop. Dr..

[78] Figueroa H., Conant N.F. (1940). The First Case of Tinea Imbricata Caused by Trichophyton Concentricum Blanchard 1896, Reported from Guatemala. Am. J. Trop. Med. Hyg..

[79] Budiastutik I., Suwarni L. (2019). Trichophyton concentricum as a cause of tinea imbricata. J. Pak. Assoc. Dermatol..

[80] Serjeantson S., Lawrence G. (1977). Autosomal recessive inheritance of susceptibility to tinea imbricata. Lancet.

[81] Jazwinska L.C., Bhatia K., Jenkias C., Serjeantson S.W. (1990). HLA class II RFLP-typing in tinea imbricata patients from Papua New Guinea. Tissue Antigens.

[82] Masons D., Marks M. (2015). Bakua: Tinea Imbricata in the Solomon Islands. Am. J. Trop. Med. Hyg..

[83] Chen Y.E., Fischbach M.A., Belkaid Y. (2018). Skin microbiota-host interactions. Nature.

[84] Sprockett D.D., Martin M., Costello E.K., Burns A.R., Holmes S.P., Gurven M.D., Relman D.A. (2020). Microbiota assembly, structure, and dynamics among Tsimane horticulturalists of the Bolivian Amazon. Nat. Commun..

[85] Weyrich L.S., Dixit S., Farrer A.G., Cooper A.J. (2015). The skin microbiome: Associations between altered microbial communities and disease. Australas. J. Dermatol..

[86] Hannigan G.D., Grice E.A. (2013). Microbial Ecology of the Skin in the Era of Metagenomics and Molecular Microbiology. Cold Spring Harb. Perspect. Med..

[87] Gordon-Thomson C., Kumari A., Tomkins L., Holford P., Djordjevic J.T., Wright L.C., Sorrell T.C., Moore G.P.M. (2009). Chitotriosidase and gene therapy for fungal infections. Cell. Mol. Life Sci..

[88] Pharma A. GRISEOFULVIN—Griseofulvin Suspension. https://dailymed.nlm.nih.gov/dailymed/fda/fdaDrugXsl.cfm?setid=af318d5d-cc39-4a63-a590-b87c50f2694f&type=display.

[89] Lee S.C., Tang M.S., Easton A.V., Devlin J.C., Chua L.L., Cho I., Moy F.M., Khang T.F., Lim Y.A.L., Loke P.N. (2019). Linking the effects of helminth infection, diet and the gut microbiota with human whole-blood signatures. PLoS Pathog..

[90] Oakley A. Terbinafine. https://dermnetnz.org/topics/terbinafine.

